# An efficient deep learning approach with frequency and channel optimization for underwater acoustic target recognition

**DOI:** 10.1038/s41598-025-12452-2

**Published:** 2025-07-28

**Authors:** Di Zeng, Shefeng Yan, Jirui Yang, Xianli Pan

**Affiliations:** 1 University of Chinese Academy of Sciences, Beijing, 101408 China; 2 Chinese Academy of Sciences, Institute of Acoustics, Beijing, 100190 China; 3 Academy for Multidisciplinary Studies, Capital Normal University, Beijing, 100048 China

**Keywords:** Ship radiated noise, Underwater acoustic, Deep learning, ResNet, Electrical and electronic engineering, Computer science

## Abstract

Ship radiated noise (SRN) recognition is challenging due to environmental noise and the broad frequency range of underwater signals. Existing deep learning models often include irrelevant frequencies and use red, green, and blue (RGB) channel configurations in convolutional networks, which are unsuitable for SRN data and computationally intensive. To address these limitations, we propose FCResNet5, a neural network optimized for SRN classification. FCResNet5 adopts a streamlined architecture that focuses on the critical frequency band and applies frequency channelization to enhance spectral representation. Its compact design achieves greater computational efficiency while maintaining comparable accuracy. Ablation studies confirm the contribution of each component, and comparative results demonstrate that FCResNet5 offers a more efficient alternative to existing models without compromising performance.

## Introduction

### Background

Ship radiated noise (SRN), generated by various onboard sources such as the service diesel generator, main engine firing rate, and blade rate harmonics due to propeller cavitation^1^, is a key signal within the complex marine environment^2^. However, the intricate and dynamic nature of this environment introduces significant interference, making it challenging to effectively extract and identify SRN features^2,3^. In addition to its role in ship classification and identification, SRN is crucial for monitoring the health of structural components and onboard systems, including motors, gears, and pumps. Accurate SRN feature extraction and identification are therefore of paramount importance in hydro-acoustic research^3^. Moreover, understanding and mitigating SRN has far-reaching implications for marine detection and defense applications^2,4,5^, playing a pivotal role in ensuring the effectiveness of naval surveillance and acoustic monitoring systems.

Advancements in signal processing and machine learning greatly enhance SRN feature extraction and identification, surpassing traditional manual detection methods based on human auditory perception^6^. Modern signal processing techniques, such as spectral and time-frequency analysis, provide more accurate and efficient interpretations of acoustic signals by extracting key features such as frequency components, amplitude, and phase. Machine learning algorithms, including Support Vector Machines (SVM), Random Forests (RF), and K-Nearest Neighbors (KNN), further improve recognition accuracy and handle the complexity of SRN data^7^. While traditional machine learning relies on manual feature engineering and is more suited for small-scale datasets with limited features^8,9^, modern deep learning models automatically learn features and offer powerful non-linear representations, particularly beneficial for larger datasets, though they require more computational resources^10,11^.

Deep learning methods, such as Convolutional Neural Networks (CNNs)^12,13^ and Deep Belief Networks (DBNs)^10^, prove especially effective in recognizing SRN by automatically extracting and learning features from raw data, significantly improving performance. Additionally, models like Recurrent Neural Networks (RNNs)^14,15^ and Long Short-Term Memory (LSTM) networks^16,17^ are well-suited for processing sequential data, making them ideal for analyzing time-series acoustic signals. LSTMs, in particular, capture long-term dependencies in the data, which is critical for recognizing patterns in SRN that may span over extended periods. More recently, Transformer models gain attention for their effectiveness in handling sequential data and capturing long-range dependencies. Leveraging an attention mechanism, transformers show superior performance in natural language processing tasks^18^, and their ability to focus on informative features while suppressing noise makes them promising for SRN recognition as well^19^.

### Motivation

Although deep learning models, particularly CNN-based approaches, improve SRN recognition by automating feature extraction and learning, several challenges remain when adapting them to the specific nature of underwater acoustic data.

The first challenge arises from the unique frequency characteristics of underwater signals. While these signals span a broad frequency range, only a narrow band around 2kHz typically contains critical information for ship classification^20–23^. Many current models process the entire bandwidth, decreasing the signal-to-noise ratio and the overall classification accuracy.

Second, CNN-based models, originally designed for image processing, often transform acoustic signals into formats resembling visual patterns, using channel configurations adapted from image data. This approach mismatches the frequency-based nature of SRN data, potentially distorting important signal characteristics and limiting the effectiveness of feature extraction.

Finally, underwater monitoring devices, especially those used in remote or deep-sea environments, face stringent constraints on power consumption and computational resources^24,25^. These devices have limited energy supply and processing capabilities, making it impractical to deploy large, resource-intensive models designed for high-performance land-based systems.

Given these limitations, there is a clear need for specialized neural network architectures tailored to SRN data. Efficient models must not only focus on the critical frequency band to exclude irrelevant information but also minimize computational load to be feasible for real-time deployment in resource-constrained underwater environments.

### Our work

In this paper, we present a novel neural network architecture: Frequency Channelization ResNet5 (FCResNet5), specifically tailored for SRN data. Our approach focuses on three key contributions:

- Targeting frequencies below 2kHz: By concentrating on the critical frequency band, we reduce the impact of irrelevant noise and interference, leading to improved recognition accuracy. This focus also reduces the overall input data volume, thereby lowering computational complexity.
- Custom network architecture for SRN data: The proposed model is designed to align with the unique characteristics of SRN signals. By leveraging frequency channels as input, it efficiently captures fine-grained information across different frequency bands. Additionally, the model adjusts the number of channels in a descending manner as the network deepens, preserving high recognition performance while optimizing resource use.
- Model compression design: To further enhance computational efficiency, we compress the model by reducing the number of ResNet layers and adjusting the kernel size, significantly decreasing parameter size and computational complexity without sacrificing performance. This compression also results in faster training time and lower resource demands, all while maintaining competitive accuracy.

We evaluate the effectiveness of these innovations through extensive experiments on the open-source Deepship dataset, demonstrating the superior performance and efficiency of FCResNet5 in SRN classification.

## Related studies

In the field of underwater acoustic target recognition, SRN plays a critical role. However, environmental noise poses significant challenges in data collection and processing. To address these challenges, lots of studies propose various signal preprocessing, feature extraction, and deep learning methods. While these approaches make substantial progress in improving recognition accuracy, they often face limitations, such as neglecting the frequency characteristics of SRN, relying on complex models, or incurring high computational costs.

**Table 1:** Comparison between existing literature and proposed method.

Aspect	Existing Literature	Proposed Method	Advantages
Preprocessing methods	FIR filtering^26^. Downsampling^19,27–29^. Pre-emphasis^30,31^.	Processing the frequencies below 2kHz.	Reduce bandwidth. Decrease computational cost. Improve accuracy.
Feature extraction	Time-domain features^6,13,27^. Time-frequency spectrograms^32,33^. Time-frequency spectrogram images^26,31^. Feature fusions^24,34–36^.	Channelizing the time-frequency spectrograms.	Improve computational efficiency.
Architectures	UALF based on ResNet50^35^. Methods expanded from ResNet18^31,37^. Methods based on various attentions^24^.	Reducing layers and reordering the channels of ResNet18.	Reduce parameter count. Improve accuracy.

### Preprocessing and feature extraction

Environmental noise, such as human activities, marine life, and multipath effects, makes data collection challenging^32,38^. Preprocessing is essential to reduce noise and interference, with common methods including FIR filtering^26,27,30^ to reduce noise and interference. However, these filters do not account for the primary frequency band of SRN. Similarly, down-sampling^19,28,29^ is often used to reduce data volume and computational complexity, but this is done without carefully considering the bandwidth characteristics of SRN. Additionally, pre-emphasis^31,39^, aiming at boosting high-frequency components, has an effect opposite to what is needed for SRN. In contrast, our approach focuses on a maximum frequency of 2kHz, which is sufficient for capturing the key features, allowing for more effective noise suppression and feature extraction. Several existing studies use CNNs to automatically extract features from underwater acoustic signals. Doan et al.^13^ propose UATC-DenseNet, a CNN that processes time-domain audio signals, demonstrating high classification accuracy on real-world passive sonar data. Yang et al.^6^ introduce an enhanced CNN with channel attention and feature fusion, achieving effective underwater signal recognition on the Shipsear and DeepShip datasets. Similarly, Li et al.^27^ develop FEM-ATNN, a feature extraction module integrating time-domain filters and time-frequency analysis with attention mechanisms to improve recognition accuracy. However the high dimensionality of time-series data can be burdensome for neural network models, making it challenging to efficiently capture key features. As a result, many studies shift towards using time-frequency to address these limitations. For example, Irfan et al.^32^ evaluate their network using time-frequency features like Mel-Frequency Cepstral Coefficients (MFCCs), wavelet packets, and Constant Q Transform (CQT), while Han et al.^33^ incorporate Mel-spectrograms, spectral contrast, and other features to enhance signal representation. Zhang et al.^34^ explore feature fusion, combining amplitude, phase, and bispectra after applying Short-Time Fourier Transform (STFT), yielding strong classification performance. Ren et al.^35^ use STFT spectrograms processed with parameterized Gabor filters, and Liu et al.^36^ employ a 3D feature set from Mel-spectrograms with delta and delta-delta features to capture the dynamics of underwater signals. Yang et al.^24^ propose a sub-band concatenated Mel (SC-Mel) spectrogram to improve feature extraction in low-frequency noise. However, these approaches primarily focus on methods derived from image processing or time-domain analysis, often overlooking the specific characteristics of acoustic signals across different frequency bands. In contrast, our approach emphasizes direct utilization of raw spectral data, specifically targeting the frequency domain aiming to provide a more effective solution for underwater acoustic signal recognition and demonstrating a unique perspective not addressed by existing methods.

### Deep learning methods

Ren et al.^35^ propose a system UALF that uses parameterized Gabor filters in its learnable front-end to extract features from underwater acoustic signals. The backend classifier is a multi-channel ResNet50 structure. The system is tested on multiple datasets under different recording conditions and scenarios. The results show that UALF outperform baseline methods, verifying its intelligence and feasibility for practical applications. Xie et al.^37^ propose a Convolution-based Mixture of Experts (CMoE) model to enhance underwater acoustic target recognition by leveraging the adaptive learning capabilities of multiple expert layers and a routing layer. They conduct comprehensive experiments and visualization analyses on three underwater acoustic databases, demonstrating that the CMoE model consistently outperforms existing advanced methods in terms of recognition accuracy. Zhu et al.^31^ present a novel underwater acoustic target recognition network known as SNANet. The SNANet is structured to extract both auxiliary and primary features within specific frequency bands and then combines these features using the proposed adaptive weights. Comprehensive experiments and ablation studies validate the effectiveness of the proposed SNANet. The experiments compare SNANet’s performance with other state-of-the-art methods and demonstrated its superiority in terms of recognition accuracy. Huan et al.^31^ introduce a novel underwater acoustic target recognition method that leverages the WA-DS decision fusion algorithm. This method replaces anomalous evidence with weighted average evidence, thereby resolving issues related to synthesizing conflicting evidence. The proposed method demonstrate a remarkable recognition accuracy on the Shipsear dataset, marking a significant improvement over single-feature recognition methods. The network structures used by these researchers all employ ResNet as the backbone. In addition, some simpler network structures also achieve very good results. Liu et al.^36^ employ a Convolutional Recurrent Neural Network (CRNN) that combines CNN for local feature extraction and LSTM for capturing temporal dependencies, suitable for time-series signal processing. The CRNN-9 model, in particular, outperforms other models, including LSTM and CNN, across all tasks. Yang et al.^24^ introduce a method coupled with a multi-domain attention mechanism integrated into a lightweight CNN model named CFTANet and evaluate the system on two open datasets, DeepShip and Shipsear. While previous methods leverage complex architectures to improve the accuracy of underwater acoustic target recognition, they often rely on highly intricate models and complex feature extraction techniques. These two factors can lead to increased computational costs and challenges in real-time processing. our approach distinguishes itself by focusing on the raw spectral data, prioritizing frequency information. This focus allows our FCResNet5 model to efficiently extract relevant features, resulting in a lightweight yet powerful architecture that significantly reduces computational complexity while maintaining high classification accuracy.

The main differences between the existing literature and the proposed method are summarized in Table 1. The proposed approach introduces improvements in preprocessing, feature extraction, and model architecture, streamlining complexity and focusing on more targeted frequency processing and network design compared to prior methods.

## Proposed method

**Fig. 1:**
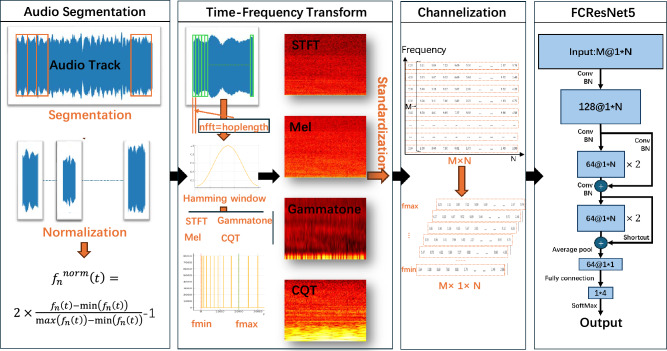
The overall processing structure of FCResNet5 method.

Our proposed method, FCResNet5, is a neural network specifically designed for SRN data. The overall structure is illustrated in Fig. 1. In the following subsections, we will systematically elaborate on the complete process in Fig. 1, including data processing, feature extraction, and model construction. Throughout the explanation, we further highlight the three main innovations of this paper.

### Preprocessing via effective bandwidth

**Fig. 2:**
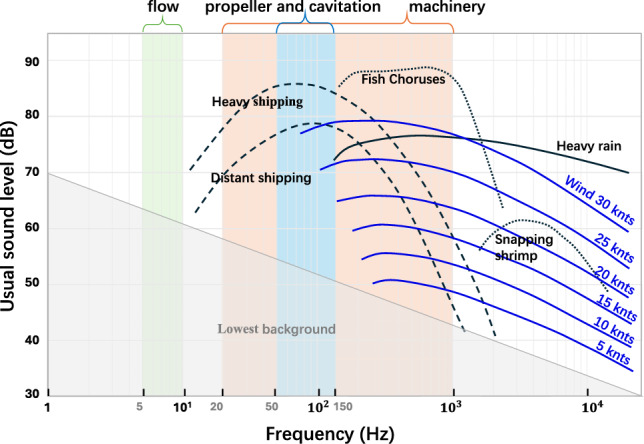
The distribution of ship and marine environmental noise^23^.

Figure 2 illustrates the spectral characteristics of various SRN sources alongside typical marine environmental noise. It can be observed that the majority of SRN energy is concentrated below 2 kHz, covering the low to mid-frequency range. This includes contributions from mechanical noise (20–1000 Hz), propeller-induced tonal components (50–150 Hz), and low-frequency hydrodynamic noise (5–10 Hz), each associated with different operational conditions and physical mechanisms^20,21,23,40^. Notably, this SRN-dominant range also overlaps with background ocean noise, which poses challenges for accurate classification^41^.

Guided by this spectral distribution, we adopt the sub-2 kHz frequency band for feature extraction, which effectively captures the primary components of SRN while reducing computational cost without compromising classification accuracy. Furthermore, this design choice is supported by our ablation studies in the *Effective Bandwidth* section, where we experimentally evaluate the impact of different frequency bands on classification performance.

### Feature extraction

For SRN classification, various feature extraction approaches have been proposed. In this study, we compare seven commonly used features, as summarized in Table 4 and discussed in Section *Comparison Between Time-Frequency and Non-Time-Frequency Features*. The results demonstrate that time-frequency features generally yield superior classification performance compared to non-time-frequency representations. Therefore, we focus on four widely adopted time-frequency features for further analysis and evaluation: STFT spectrogram, Mel spectrogram, CQT spectrogram, and Gamma-tone spectrogram. The extraction methods for these features are detailed below.

#### STFT spectrogram

The Short-Time Fourier Transform (STFT) decomposes a time-domain signal into short-time segments using a sliding window, then applies the Fourier transform to each segment to extract localized frequency information. The STFT is defined as:

1 $$\begin{aligned} X(t, f) = \int _{-\infty }^{\infty } x(\tau ) w(\tau - t) e^{-j 2 \pi f \tau } , d\tau \end{aligned}$$

where $x(\tau )$ denotes the original signal, $w(\tau - t)$ is the analysis window centered at time *t*, and *f* is the frequency. *X*(*t*, *f*) represents the complex-valued STFT of the signal, encoding its time-frequency energy distribution.

#### Mel spectrogram

The Mel spectrogram projects the STFT power spectrum onto a perceptually motivated Mel frequency scale, which models the nonlinear sensitivity of human hearing. The Mel frequency scale is defined by:

2 $$\begin{aligned} \text {Mel}(f) = 2595 \times \log _{10} \left( 1 + \frac{f}{700} \right) \end{aligned}$$

This mapping is approximately linear below 1 kHz and logarithmic above. The Mel spectrogram $S_{\text {mel}}(t, m)$ is then computed by applying a bank of Mel filters to the STFT power spectrum:

3 $$\begin{aligned} S_{\text {mel}}(t, m) = \sum _{k=1}^{N} |X(t, f_k)|^2 H_m(f_k) \end{aligned}$$

where $|X(t, f_k)|^2$ is the power spectrum at frequency bin $f_k$, and $H_m(f_k)$ is the response of the *m*-th Mel filter. The result $S_{\text {mel}}(t, m)$ represents the energy distribution over Mel frequency bands at each time frame.

#### Gamma-tone spectrogram

The Gamma-tone spectrogram employs a filter bank composed of Gamma-tone filters, which are widely used to simulate the frequency selectivity of the human auditory system. The impulse response of a Gamma-tone filter is given by:

4 $$\begin{aligned} g(t) = t^{n-1} e^{-2 \pi b t} \cos (2 \pi f_c t + \phi ) \end{aligned}$$

Here, $t^{n-1}$ defines the envelope shape, $e^{-2 \pi b t}$ controls the filter’s bandwidth, and $\cos (2 \pi f_c t + \phi )$ is the sinusoidal carrier centered at frequency $f_c$ with phase offset $\phi$. The output of the Gamma-tone filter bank provides a biologically inspired time-frequency representation of the signal.

#### CQT Spectrogram

The Constant-Q Transform (CQT) performs frequency analysis using a logarithmically spaced set of frequency bins, providing high resolution for low frequencies and finer time resolution for high frequencies. It is formulated as:

5 $$\begin{aligned} X_{\text {CQT}}(k, t) = \frac{1}{N_k} \sum _{n=0}^{N_k - 1} x[n] w\left( n - \frac{N_k}{2}\right) e^{-j 2 \pi \frac{k}{N_k} n} \end{aligned}$$

where $N_k$ is the window length for the *k*-th frequency bin, *w*[*n*] is the analysis window, and *x*[*n*] is the input signal. $X_{\text {CQT}}(k, t)$ denotes the CQT coefficient, reflecting the spectral content of the signal with constant-Q bandwidths.

### Tailored network architecture

In this work, we propose a network architecture specifically designed to align with the unique characteristics of SRN data. The design centers around two key strategies: leveraging frequency channels to align with the structure of time-frequency features and employing a descending channel structure to improve feature extraction performance.

#### Frequency channelization

In CNNs, the concept of channels is a fundamental design element, particularly suited for image processing tasks. For instance, in RGB images, the input is composed of three distinct channels: red, green, and blue, each representing different color components of the image. This multi-channel structure allows CNNs to effectively process and extract useful features from complex visual data.

**Fig. 3:**
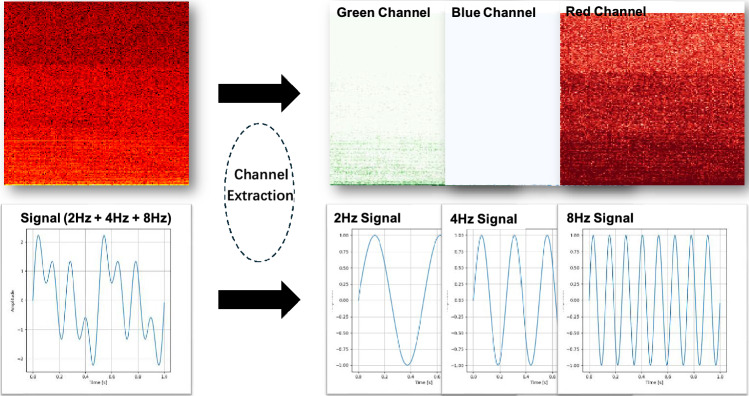
The core concept of frequency channelization.

However, in signal processing, the input data, such as time-frequency representations, does not have the concept of channels similar to the RGB channels in images. Recognizing that signals can be decomposed into a series of sine waves at different frequencies, we propose an alternative approach: treating the frequency dimension as the channel dimension within CNNs. The core concept of this method, illustrated in Fig. 3, allows the network to process signals in a way that reflects their inherent frequency composition. Just as RGB channels represent distinct components of an image, frequency channels represent different frequency bands within the signal, enabling more efficient feature extraction from SRN data.

#### Descending channel structure

**Fig. 4:**
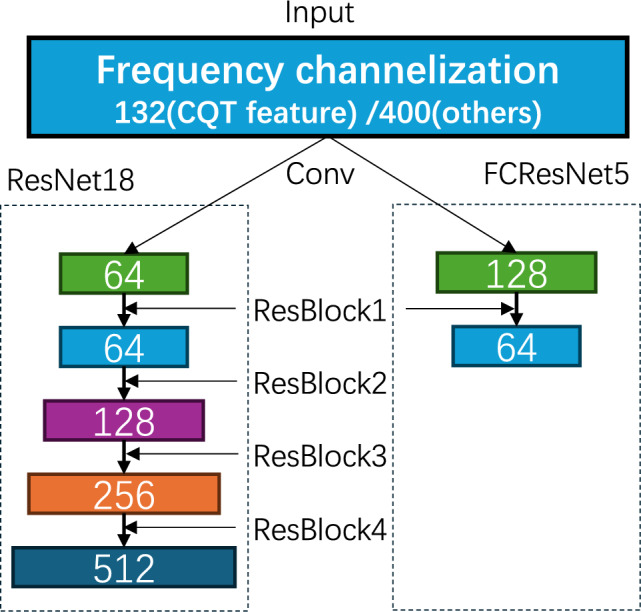
The comparison of the number of channels and layers between ResNet18 and FCResNet5.

Building on the frequency channelization approach discussed in the previous subsection, we find that directly employing ResNet18 is not the most suitable option. Specifically, as illustrated in Fig. 4, the input feature maps have much more channels (132 for the CQT feature and 400 for others). In the original ResNet18 structure (left panel of Fig. 4), the number of channels initially decreases and then increases progressively, which does not align well with the frequency channelization setup.

To address this issue, we modify the channel configuration by adopting a descending structure, where the number of channels gradually decreases as the network deepens. This design reduces feature dimensionality in a more logical manner, as depicted in the right panel of Fig. 4. The importance of this descending channel structure in improving recognition accuracy will be validated by the experiments in Section 5, which highlight the significant impact of this adjustment on overall model performance.

### Model compression design

In addition to enhancing computational efficiency through bandwidth adjustment and aligning the network with signal characteristics, we further reduce complexity by compressing the network structure. Specifically, we propose compressing the ResNet18 architecture by reducing the number of layers and adjusting the kernel size, all while maintaining model performance. To evaluate the impact of these modifications, we conduct experiments to compare network performance under different configurations, specifically varying the number of ResBlock groups. Key metrics, including accuracy, parameters, MFLOPs, and average training time, are assessed for each configuration. Based on a comprehensive assessment of these factors, we find that using a single ResBlock group provides a good balance between computational efficiency and recognition performance. Consequently, our final model design incorporates only one ResBlock group, as depicted in Fig. 4, where the numbers represent the change in the number of feature channels. In FCResNet5, the initial convolution reduces the input channels, followed by a single ResBlock that further decreases the channels, creating a simplified architecture with fewer layers compared to ResNet18. This streamlined design reduces model complexity while still achieving competitive accuracy, making it more efficient for practical applications.

**Fig. 5:**
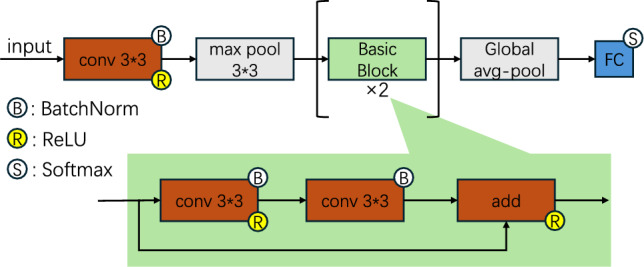
Architecture diagram of FCResNet5.

### Overall structure

Incorporating the settings discussed above, we present the overall structure of our FCResNet5, as shown in Fig. 5. It consists of one convolutional layer, one ResBlock, and one classification layer, with the number of channels decreasing sequentially. Detailed parameters are provided in Table 2.

**Table 2:** Detailed parameters of FCResNet5.

Layer	Parameters
Conv	Conv2d: In=400(132 for CQT), Out=128, Kernel=3, Stride=1, Padding=1 BatchNorm2d: NumFeatures=128 ReLU() MaxPool: Kernel=3, Stride=1, Padding=1
ResBlock	Conv: Conv2d: In=128, Out=64, Kernel=3, Stride=1, Padding=1 BatchNorm2d: NumFeatures=64 ReLU() Conv2d: In=64, Out=64, Kernel=3, Stride=1, Padding=1 BatchNorm2d: NumFeatures=64
	Shortcut: Conv2d: In=128, Out=64, Kernel=1, Stride=1, Padding=1 BatchNorm2d: NumFeatures=64
	ReLU()
	Conv: Conv2d: In=64, Out=64, Kernel=3, Stride=1, Padding=1 BatchNorm2d: NumFeatures=64 ReLU() Conv2d: In=64, Out=64, Kernel=3, Stride=1, Padding=1 BatchNorm2d: NumFeatures=64
	ReLU()
Classification	AdaptiveAvgPool2d(1, 1) Linear: In=64, Out=4

**Algorithm 1:**
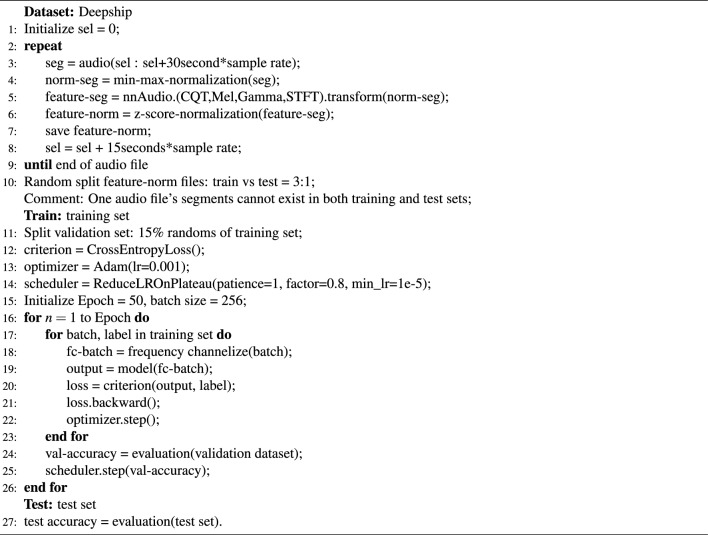
Pseudocode for FCResNet5.

The pseudocode for the overall process of our FCResNet5 is provided in Algorithm 1. First, audio files are divided into 30-second segments, with a 15-second overlap between consecutive segments. Each segment undergoes min-max normalization to ensure consistency in value range. Time-frequency features are then extracted from each segment using multiple transformations, including STFT, Mel, Gamma-tone, and CQT. The resulting feature segments are further normalized using z-score normalization. Following feature extraction, the dataset is divided into training and test sets at a 3:1 ratio and we need to ensure that no segment from one audio file appears in both sets simultaneously. During training, 15% of the training set is reserved for validation. The model is trained using the cross-entropy loss function and the Adam optimizer. A learning rate scheduler, ReduceLROnPlateau, adjusts the learning rate based on validation accuracy. The model is trained for 50 epochs with a batch size of 256.

## Experiment

### Dataset

In our experiment, we use the open-source DeepShip dataset^32^, which is designed for marine vessel recognition. The data are collected using hydrophones deployed at the Strait of Georgia Delta node over a 29-month period, from May 2, 2016 to October 4, 2018. The recordings take place under realistic maritime conditions, with timestamps and vessel identities obtained through the Automatic Identification System (AIS). To ensure data clarity, only acoustic signals emitted by a single vessel within a 2 km radius of the hydrophone are retained, and data collection is paused when the vessel moves beyond this range.

**Fig. 6:**
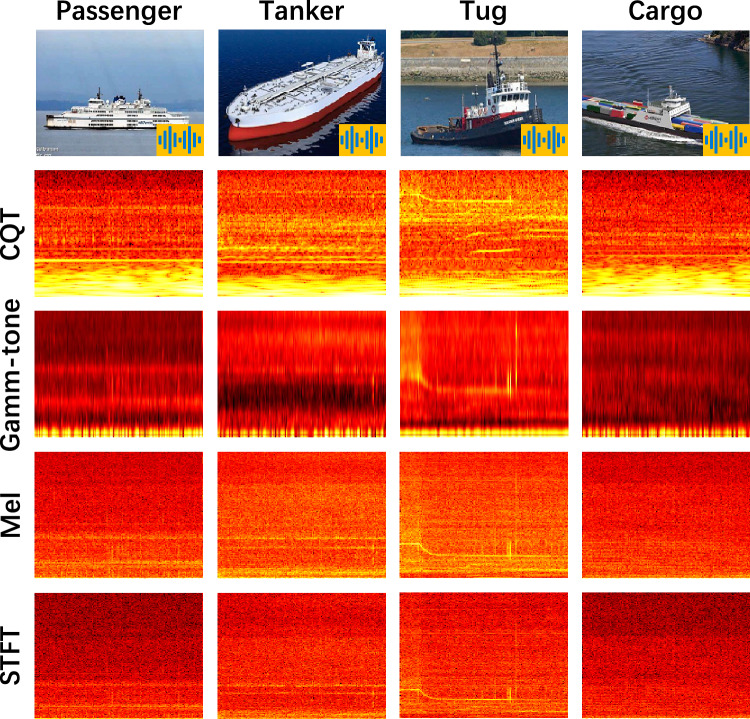
Four classes of DeepShip and the four spectrograms.

The dataset encompasses recordings from four distinct classes of ships, namely oil tankers, tugboats, passenger ships, and cargo ships, as shown in the first row of Fig. 6. It contains 613 recordings from 265 individual vessels. The total recording duration is approximately 47 hours and 4 minutes, with individual clips ranging from 6 to 1530 seconds depending on vessel type, location, and motion. All signals are sampled at 32kHz, and the selected recordings ensure a relatively balanced duration across the four classes. Figure 6 also displays representative spectrograms of each class extracted using four typical time–frequency analysis methods: CQT, Gamma-tone, Mel, and STFT.

### Dataset splitting

In this section, we detail the methodology used to preprocess the audio data and prepare the dataset for training and evaluation. This includes the process of audio track segmentation, as well as the strategy for splitting the data into training and test sets, both of which are crucial for ensuring the accuracy and consistency of our model’s performance.

#### Audio track splitting

In this study, we employ a segmentation method to process the audio tracks from the DeepShip dataset. Each audio track is divided into frames of 30 seconds in duration. To ensure continuity and capture sufficient contextual information, we implement an overlap of 15 seconds between consecutive frames. This approach allows each frame to retain part of the preceding and succeeding audio content, which is crucial for tasks such as classification or recognition. To avoid the introduction of artificial features, any segment shorter than 30 seconds is excluded from the analysis. This ensures that only sufficiently long segments, which can provide meaningful information, are considered. To formally describe the process, let the time series data be represented by *x*(*t*), where *t* is the time index, then the *n*-th frame $f_n(t)$ can be expressed as $f_n(t) = x(t + (n - 1) \times 15s)$ where *n* is the frame index.

After framing the time series data, each frame undergoes a normalization process. This normalization scales the data within each frame to fall within the range [-1, 1]. Let $f_n$ denote the *n*-th frame and $f_n^{norm}$ represent the normalized frame. The normalization is performed as follows:

6 $$\begin{aligned} f_n^{\text {norm}}(t) = 2 \times \frac{f_n(t) - \min (f_n(t))}{\max (f_n(t)) - \min (f_n(t))} - 1 \end{aligned}$$

where $\max (f_n(t))$ and $\min (f_n(t))$ are the maximum and minimum values of the frame $f_n(t)$ respectively.

#### Training and test set splitting

In this study, we randomly divide the dataset with a training set comprising approximately 75% of the data and a test set comprising approximately 25% of the data. Additionally, we ensure that the proportion of each class in the training and test sets remain consistent with the overall class distribution across all groups. To maintain the integrity of the dataset and avoid introducing bias, audio tracks generated from the same audio file are kept exclusively in either the training or testing dataset, ensuring that correlated data does not appear in both sets.

### Feature extraction through nnAudio

In our study, we utilize nnAudio for feature extraction, processing all relevant features. To ensure optimal frequency resolution, we set the FFT frame length and frame shift to 200 ms, resulting in no overlap between frames. The frequency range is restricted to 1 Hz to 2 kHz, and a Hamming window function is applied. The detailed parameters are provided in Table 3. Additionally, Fig. 6 provides visual examples of the four ship classes, each accompanied by corresponding time–frequency spectrograms generated using the adopted feature extraction methods.

**Table 3:** Feature extraction parameter.

nnAudio	fmin	fmax	bins	nfft/ hoplength	Window	octave_bins	Dimension
STFT Mel Gamma-tone	1Hz	2kHz	400	0.2*fs	Hamming	–	400 * 150
CQT	1Hz	2kHz	–	0.2*fs	Hamming	12	132 * 150

* fs: 32 kHz sampling rate.

### Training parameters

During training, we utilize the Adam optimizer along with the ReduceLROnPlateau learning rate scheduler, starting with an initial learning rate of 0.001. The learning rate decay factor is set to 0.8 with a step size of 1 epoch, and the minimum learning rate is set to 1e-5. All experiments are conducted on a system equipped with an Intel i9-10900K CPU and an NVIDIA GeForce RTX 3060 GPU with 12 GB of VRAM.

The implementation is based on PyTorch 2.2.2 and nnAudio 0.3.3, running in a Python 3.12.2 environment. We do not apply fixed random seeds in order to simulate the average-case behavior, and all experiments are repeated ten times with different random splits. The final results are reported as the mean values across these ten runs.

## Results

In this section, we conduct two experiments to validate the efficiency and superiority of our proposed method. The first is an ablation experiment to verify the impact of key model components. The second is a comparative experiment to evaluate our method against state-of-the-art approaches in terms of accuracy and efficiency.

### Ablation experiment

In this section, we conduct a comprehensive ablation study to investigate the key design factors contributing to the performance of FCResNet5. Specifically, we compare time-frequency and non-time-frequency input features, assess the influence of frequency bandwidth selection, examine the effect of window overlap during feature extraction, evaluate the role of frequency channelization, and explore suitable network architectures. These analyses collectively demonstrate how FCResNet5 achieves a balance between accuracy and computational efficiency, making it well-suited for real-world ship-radiated noise classification.

#### Comparison Between Time-Frequency and Non-Time-Frequency Features

To evaluate the effectiveness of different input representations for ship-radiated noise classification, we conduct a comparative experiment using ResNet18 across seven feature types. These include four time-frequency features (STFT, Mel, CQT, and Gamma-tone) and three non-time-frequency features (MFCC, Wavelet, and Cepstrum). Each feature is evaluated using five randomly generated data splits, and the results are averaged over 10 repeated runs. The average classification accuracies are summarized in Table 4.

**Table 4:** Average classification accuracy (%) of ResNet18 using seven feature representations.

Feature type	Average accuracy (%)
STFT	72.38
Mel	71.20
CQT	66.36
Gamma-tone	64.38
MFCC	65.06
Wavelet	50.86
Cepstrum	53.75

As shown in Table 4, the time-frequency representations consistently outperform the non-time-frequency counterparts. Among them, STFT achieves the highest average accuracy (72.38%), followed closely by Mel (71.20%), while Wavelet and Cepstrum exhibit the lowest performance. These findings suggest that time-frequency features preserve more discriminative information crucial for ship-radiated noise classification.

**Fig. 7:**
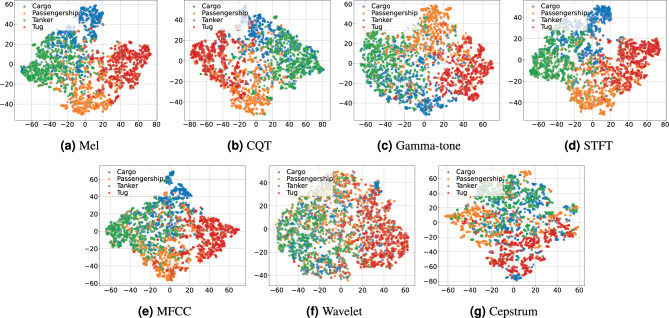
t-SNE visualizations of learned features using seven different feature types: Mel, CQT, Gamma-tone, STFT, MFCC, Wavelet, and Cepstrum.

To further evaluate feature separability, we visualize the t-SNE embeddings of the extracted features in Fig. 7. Subfigures (a) to (d), which correspond to the time-frequency representations, exhibit more distinct and compact clusters, reflecting stronger inter-class separability. In contrast, the non-time-frequency features in subfigures (e) to (g), particularly Wavelet and Cepstrum, show more diffuse and overlapping distributions, indicating limited discriminative capability. These qualitative observations are consistent with the classification accuracy results summarized in Table 4.

Based on this analysis, we adopt the four time-frequency features (STFT, Mel, CQT, and Gamma-tone) as the primary input representations for subsequent experiments.

#### Effective bandwidth

In this section, we validate the rationale behind selecting the 2kHz bandwidth. In our experiments, we use ResNet18 as the classification method to test the impact of different upper and lower frequency limits on the model’s performance.

**Fig. 8:**
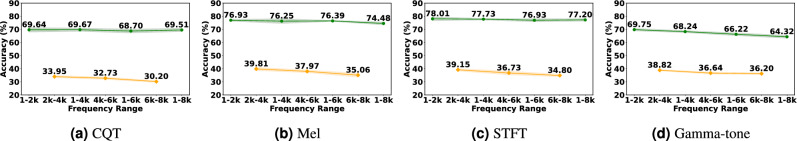
Accuracy (mean ± std) of different frequency ranges on four different types of features. The green lines represent the results under different maximum frequency cutoffs, while the orange lines correspond to different frequency band segments.

The experimental results are illustrated in Fig. 8 and Table 5, which jointly present the classification accuracy (mean ± std over 10 trials) of ResNet18 under various frequency input configurations across four spectral features: CQT, Mel-spectrogram, STFT, and Gamma-tone. Figure 8 visualizes the accuracy trends for different maximum frequency cutoffs (green lines) and segmented frequency bands (orange lines), while Table 5 reports the detailed numerical values.

As shown by the green line, the model’s classification performance generally decreases as the bandwidth range increases, indicating that increasing the upper bandwidth limit does not improve the model’s performance but instead introduces interference. Furthermore, the yellow line also shows that higher bandwidth ranges correspond to lower classification accuracy, suggesting that these ranges likely do not contain useful signals for classification, but rather unwanted noise. Thus, this experiment confirms the rationale of our setting by limiting the data bandwidth to within 2 kHz.

**Table 5:** Accuracy (mean ± std) of FCResNet5 under different frequency range and band settings across four types of spectral features.

Feature	1–2k	2k–4k	1–4k	4k–6k	1–6k	6k–8k	1–8k
CQT	69.64 ± 1.78	33.95 ± 0.87	**69.67** ± **1.29**	32.73 ± 0.97	68.70 ± 1.53	30.20 ± 0.78	69.51 ± 1.31
Mel	**76.93** ± **0.82**	39.81 ± 1.25	76.25 ± 1.84	37.97 ± 1.10	76.39 ± 0.47	35.06 ± 1.55	74.48 ± 1.17
STFT	**78.01** ± **1.47**	39.15 ± 0.94	77.73 ± 0.68	36.73 ± 1.50	76.93 ± 1.21	34.80 ± 1.01	77.20 ± 1.01
Gamma	**69.75** ± **0.98**	38.82 ± 0.64	68.24 ± 0.84	36.64 ± 0.85	66.22 ± 1.08	36.20 ± 0.74	64.32 ± 1.15

The best results are highlighted in bold.

#### Analyzing Window Overlap in Spectral Feature Extraction

To investigate the influence of windowing strategies on model performance and training efficiency, we conduct an ablation study on the use of overlapping windows during time-frequency feature extraction. In this experiment, we fix the window length at 200 ms and vary the overlap ratio between adjacent frames. Specifically, we consider four overlap settings: 0% (no overlap), 25%, 50%, and 75%, which correspond to hop sizes of 200 ms, 150 ms, 100 ms, and 50 ms, respectively. For each setting, the model is trained and evaluated 10 times using different random seeds. We report the average classification accuracy and standard deviation across ten repeated runs, as shown in Table 6.

**Table 6:** Performance comparison under different overlap settings (accuracy: mean ± std; training time in seconds).

Feature	No overlap	25%	50%	75%
STFT	78.03 ± 1.21 **@364.1s**	77.14 ± 1.34 @545.9s	78.60 ± 0.88 @691.7s	**78.78** ± **1.16** @1229.4s
MEL	76.29 ± 0.90 **@366.0s**	**77.86** ± **1.22** @484.4s	77.31 ± 1.53 @625.4s	76.73 ± 1.39 @1055.4s
Gamma-tone	69.61 ± 1.02 **@360.2s**	69.99 ± 1.15 @479.6s	69.61 ± 0.78 @607.2s	**71.46** ± **1.24** @1064.6s
CQT	69.12 ± 1.42 **@102.7s**	**69.65** ± **1.71** @131.0s	68.87 ± 1.50 @269.0s	68.48 ± 1.41 @482.6s

The best results are highlighted in bold.

The results indicate that applying overlap generally improves classification performance for most feature types. For example, STFT accuracy improves from 78.03% (±1.21) without overlap to 78.78% (±1.16) with 75% overlap. A similar pattern is observed for the Gamma-tone feature, which increases from 69.61% (±1.02) to 71.46% (±1.24). MEL features also show moderate gains with overlap, although the improvement is less consistent. In contrast, the CQT feature shows a decrease in accuracy when the overlap exceeds 25%.

However, the benefit of overlapping comes at the cost of increased training time. Due to the denser frame segmentation, total training time grows significantly as overlap increases. For instance, STFT training time increases from 364 s at 0% overlap to 1229 s at 75% overlap. This highlights a trade-off between performance and computational cost. While overlapping can marginally enhance performance, particularly for STFT and Gamma-tone, it also leads to substantially higher resource consumption. As such, the no-overlap setting is adopted in our default configuration to ensure a good balance between accuracy and efficiency.

#### Effectiveness of frequency channelization

**Fig. 9:**
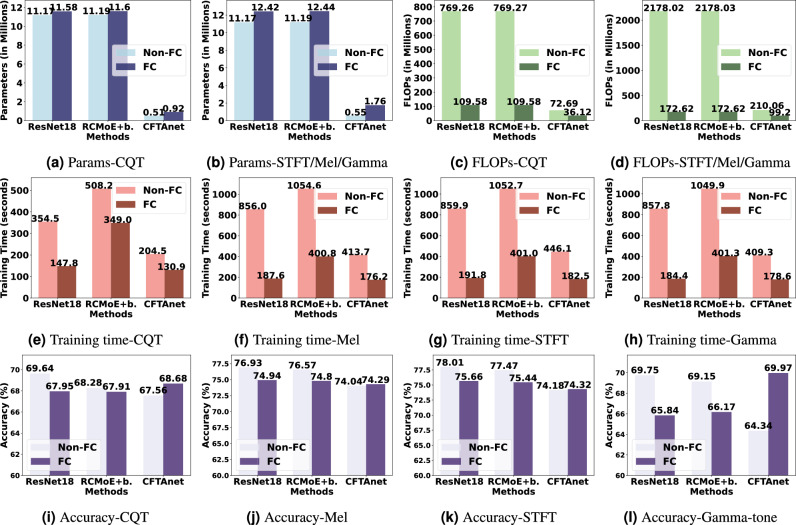
Figures of parameters (**a**-**b**), flops (**c**-**d**), training time (**e**-**h**), Accuracy (**i**-**l**) comparison on three methods with/without frequency channelization.

In this section, we evaluate the effectiveness of Frequency Channelization (FC) by applying it to three representative models: ResNet18, RCMoE-balance, and CFTAnet. The “FC” suffix indicates that the model employs frequency channelization. A comprehensive comparison of model complexity (parameters and MFLOPs), average training time, and classification accuracy (mean ± std over 10 trials) before and after applying FC is presented in Table 7 and visualized in Fig. 9.

**Table 7:** Comparison of model complexity (parameters and FLOPs), training time, and accuracy between FC and Non-FC versions of different methods across spectral features.

Metric	ResNet18			RCMoE-balance			CFTAnet		
	Non-FC	FC	$\Delta$	Non-FC	FC	$\Delta$	Non-FC	FC	$\Delta$
*Params (Million)*									
CQT	**11.17**	11.58	+0.41	**11.19**	11.60	+0.41	**0.51**	0.92	+0.41
STFT/Mel/Gamma-tone	**11.17**	12.42	+1.25	**11.19**	12.44	+1.25	**0.55**	1.76	+1.21
*FLOPs (Million)*									
CQT	769.26	**109.58**	−659.68	769.27	**109.58**	−659.69	72.69	**36.12**	−36.57
STFT/Mel/Gamma-tone	2178.02	**172.62**	−2005.4	2178.03	**172.62**	−2005.4	210.06	**99.2**	−110.86
*Training Time (Seconds)*									
CQT	354.5	**147.8**	−206.7	508.2	**349.0**	−159.2	204.5	**130.9**	−73.6
Mel	856.0	**187.6**	−668.4	1054.6	**400.8**	−653.8	413.7	**176.2**	−237.5
STFT	859.9	**191.8**	−668.1	1052.7	**401.0**	−651.7	446.1	**182.5**	−263.6
Gamma-tone	857.8	**184.4**	−673.4	1049.9	**401.3**	−648.6	409.3	**178.6**	−230.7
*Accuracy (%)*									
CQT	**69.64**	67.95	−1.69	**68.28**	67.91	−0.37	67.56	**68.68**	+1.12
Mel	**76.93**	74.94	−1.99	**76.57**	74.80	−1.77	74.04	**74.29**	+0.25
STFT	**78.01**	75.66	−2.35	**77.47**	75.44	−2.03	74.18	**74.32**	+0.14
Gamma-tone	**69.75**	65.84	−3.91	**69.15**	66.17	−2.98	64.34	**69.97**	+5.63

The best results are highlighted in bold.

Table 7 and Fig. 9 demonstrate that introducing frequency channelization leads to a marginal increase in parameter count, ranging from approximately 0.4 to 1.25 million across all models and feature types. Despite this minor increase in model size, the reduction in computational cost is substantial. For example, the number of FLOPs drops by over 90% for ResNet18 and RCMoE-balance under all feature types, and by more than 50% for CFTAnet. These reductions translate into significantly lower training times. ResNet18 sees the largest improvement, with training time reduced by more than 660 seconds, while CFTAnet also benefits with reductions of over 230 seconds across most settings. These results confirm that FC enhances computational efficiency considerably, while introducing only a modest increase in model complexity.

In terms of classification accuracy, FC yields consistent performance improvements for CFTAnet across all spectral features. Notably, the gain is particularly pronounced for the Gamma-tone input, where the accuracy improves by 5.63%. In contrast, ResNet18 and RCMoE-balance exhibit slight drops in accuracy (generally less than 2–4%), suggesting that the standard ResNet18 backbone may not be optimally aligned with frequency-channelized inputs. Since RCMoE-balance also builds on ResNet18, this pattern reinforces the hypothesis that architectural compatibility plays a key role in leveraging the benefits of FC.

Overall, these results confirm that frequency channelization significantly enhances training efficiency while preserving, and in some cases improving, classification accuracy, particularly when paired with lightweight models such as CFTAnet. This highlights the importance of designing network architectures that are structurally adapted to frequency-segmented inputs.

#### Exploring optimal network architectures

**Fig. 10:**
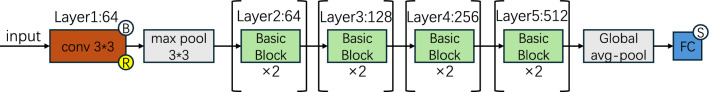
The network architecture of ResNet18.

In this section, we aim to explore network architectures that are better suited for frequency channelization. The network architecture of ResNet18 is given in Fig. 10, where the numbers 64, 128, 256, and 512 represent the feature channels generated at each layer. The diagram shows a progressive increase in the number of channels across the layers. However, after applying frequency channelization, the number of channels is 132 for CQT and 400 for other features, both significantly higher than the initial 64 channels after the first convolutional layer of ResNet18. This suggests that our input features undergo a rapid decrease in channel numbers before gradually increasing again, which we suspect may negatively impact the performance of frequency channelization. To further investigate, we conduct comparative experiments between descending and ascending channel configurations and evaluate different layer depths to assess their impact on performance.

In our experiments, the initial convolution layer is treated as the first layer, with the subsequent four groups of residual blocks treated as layers 2, 3, 4, and 5. The number of channels is adjusted uniformly for each group, and network depth is controlled by reducing the number of residual blocks. We use STFT as input features and set the maximum channel number to 256, since the final channel number in ResNet18 (512) exceeds our input’s channel number (400). Tables 8 and 9 compare accuracy (mean ± std over 10 trials), parameters, MFLOPs and average training time under descending and ascending channel configurations, respectively. In the table, the rows where the models achieved relatively higher accuracy are highlighted in bold to emphasize their performance.

The results show that the descending channel configuration consistently achieves higher accuracy than the ascending configuration under the same conditions, with the difference becoming more pronounced as the number of layers increases. However, the descending configuration requires substantially more parameters and higher computational complexity, suggesting a trade-off between accuracy and efficiency.

**Table 8:** Table of accuracy, params, FLOPs, and average training time for different maximum channels and layers with ascending. Accuracy greater than 76% is highlighted.

#Layers	#Channels	Accuracy(%) (mean ± std)	#Params(M)	MFLOPs	Time-train(s)
2	128$\rightarrow$256	75.86 ± 0.50	4.610	267.911	199.6
3	64$\rightarrow$128$\rightarrow$256	75.43 ± 0.75	3.881	153.904	183.9
4	32$\rightarrow$64$\rightarrow$128$\rightarrow$256	74.94 ± 0.93	3.385	82.995	183.9
5	16$\rightarrow$32$\rightarrow$64$\rightarrow$128$\rightarrow$256	73.08 ± 1.06	3.105	43.012	183.8
2	64$\rightarrow$128	**76.06** ± **0.72**	1.781	114.033	192.7
3	32$\rightarrow$64$\rightarrow$128	74.96 ± 0.99	1.285	62.010	182.2
4	16$\rightarrow$32$\rightarrow$64$\rightarrow$128	73.35 ± 1.26	1.005	32.549	184.2
5	8$\rightarrow$16$\rightarrow$32$\rightarrow$64$\rightarrow$128	69.84 ± 1.03	0.856	16.640	184.2
2	32$\rightarrow$64	75.68 ± 1.01	0.759	52.036	167.9
3	16$\rightarrow$32$\rightarrow$64	74.77 ± 1.39	0.479	24.366	175.7
4	8$\rightarrow$16$\rightarrow$32$\rightarrow$64	71.66 ± 0.85	0.330	14.015	167.6
5	4$\rightarrow$8$\rightarrow$16$\rightarrow$32$\rightarrow$64	66.68 ± 1.89	0.254	7.103	171.9
2	16$\rightarrow$32	74.14 ± 0.81	0.347	24.773	173.9
3	8$\rightarrow$16$\rightarrow$32	71.17 ± 1.49	0.198	12.701	174.4
4	4$\rightarrow$8$\rightarrow$16$\rightarrow$32	68.11 ± 0.96	0.122	6.152	167.1
5	2$\rightarrow$4$\rightarrow$8$\rightarrow$16$\rightarrow$32	56.95 ± 3.56	0.089	3.083	165.1
2	8$\rightarrow$16	71.98 ± 1.37	0.163	12.207	160.7
3	4$\rightarrow$8$\rightarrow$16	69.68 ± 3.89	0.089	6.107	161.7
4	2$\rightarrow$4$\rightarrow$8$\rightarrow$16	59.11 ± 3.07	0.050	3.083	157.4

**Table 9:** Table of accuracy, params, FLOPs, and average training time for different maximum channels and layers with descending. Accuracy greater than 76% is highlighted.

#Layers	#Channels	Accuracy(%) (mean ± std)	#Params(M)	MFLOPs	Time-train(s)
1	256	73.18 ± 0.41	5.019	376.340	199.0
2	256$\rightarrow$128	**76.68** ± **0.71**	5.790	405.626	200.1
3	256$\rightarrow$128$\rightarrow$64	**76.59** ± **0.74**	5.983	409.289	200.1
4	256$\rightarrow$128$\rightarrow$64$\rightarrow$32	**76.59** ± **0.76**	6.031	409.772	200.8
5	256$\rightarrow$128$\rightarrow$64$\rightarrow$32$\rightarrow$16	**76.70** ± **0.84**	6.043	409.833	201.0
1	128	72.61 ± 0.54	2.510	188.170	192.7
2	128$\rightarrow$64	**76.27** ± **0.58**	2.702	195.498	186.3
3	128$\rightarrow$64$\rightarrow$32	**76.68** ± **0.58**	2.751	196.415	187.7
4	128$\rightarrow$64$\rightarrow$32$\rightarrow$16	**76.55** ± **0.63**	2.763	196.445	188.9
5	128$\rightarrow$64$\rightarrow$32$\rightarrow$16$\rightarrow$8	**76.57** ± **0.65**	2.766	196.551	188.5
1	64	71.88 ± 0.68	1.255	95.920	162.2
2	64$\rightarrow$32	**76.34** ± **0.53**	1.303	95.920	166.1
3	64$\rightarrow$32$\rightarrow$16	75.68 ± 1.08	1.315	96.150	179.0
4	64$\rightarrow$32$\rightarrow$16$\rightarrow$8	75.46 ± 1.04	1.318	96.180	175.2
5	64$\rightarrow$32$\rightarrow$16$\rightarrow$8$\rightarrow$4	74.10 ± 1.26	1.319	96.184	177.9
1	32	71.13 ± 0.49	0.627	47.043	170.4
2	32$\rightarrow$16	75.17 ± 1.16	0.640	47.503	167.6
3	32$\rightarrow$16$\rightarrow$8	75.62 ± 0.86	0.643	47.561	169.4
4	32$\rightarrow$16$\rightarrow$8$\rightarrow$4	72.46 ± 5.91	0.643	47.568	159.5
5	32$\rightarrow$16$\rightarrow$8$\rightarrow$4$\rightarrow$2	63.18 ± 4.21	0.644	47.569	168.5
1	16	71.23 ± 0.46	0.314	23.521	162.5
2	16$\rightarrow$8	73.42 ± 0.91	0.317	23.637	163.7
3	16$\rightarrow$8$\rightarrow$4	73.70 ± 1.12	0.318	23.652	163.1
4	16$\rightarrow$8$\rightarrow$4$\rightarrow$2	67.22 ± 4.68	0.318	23.654	153.0

From Table 9, we conclude that maximum channel numbers of 256 and 128 yield the best accuracy. Furthermore, when the number of layers exceeds 2, accuracy differences are minimal. Therefore, to balance accuracy and efficiency, we select the 128$\rightarrow$64 configuration for further experiments, considering that the CQT feature input has 132 channels.

In analyzing the initial convolution layer, we find that its kernel size has a significant impact on network performance. The original ResNet18 uses a kernel size of 7 with a stride of 2. We adjust the stride to 1 and compare the impact of different kernel sizes on network performance.

**Fig. 11:**
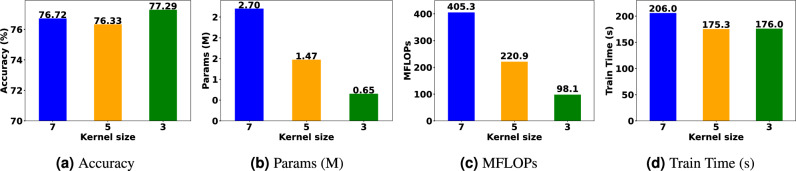
Comparison of different kernel sizes. (**a**) accuracy, (**b**) number of parameters, (**c**) computational complexity measured in MFLOPs, and (**d**) average training time.

Figure 11 shows the comparison results of different kernel sizes for accuracy (mean over 10 trials), parameters, MFLOPs, and average training time. We can see that kernel size of 3 achieve the best performance with the lowest parameters and complexity.

Based on these results, we name our optimal network FCResNet5. This network includes 5 layers: an initial convolution layer and one residual block, with each residual block comprising two residual networks, and each residual network containing two convolutional layers. The name FCResNet5 reflects its simplified ResNet structure, lightweight design, and frequency channelization.

### Comparative experiment

To comprehensively assess the effectiveness of the proposed FCResNet5, we design two complementary comparative experiments. The first focuses on evaluating classification performance using four types of time-frequency spectral features: STFT, Mel, CQT, and Gamma-tone. The second assesses the robustness of different models under varying signal-to-noise ratio (SNR) levels, simulating real-world scenarios with degraded acoustic quality. These two perspectives jointly offer a holistic evaluation of both discriminative capacity and noise resilience.

#### Performance across spectral features

In the first part of the comparative study, we evaluate the classification performance of FCResNet5 against several representative methods, including RCMoE-balance^37^, CFTAnet^24^, and the widely used backbone network ResNet18^8,26,37,42^. The dataset is divided into five distinct training and testing folds, as detailed in Table 10, ensuring that the evaluation covers diverse data distributions. We report the average classification accuracy along with standard deviation over ten independent runs, as well as model complexity metrics including parameter count, MFLOPs, and average training time. Furthermore, to support the reliability of the comparisons, we perform statistical significance tests using independent two-sample t-tests ($n=50$), with a Bonferroni-corrected threshold of $p=0.0167$.

**Table 10:** Dataset split summary.

Datasets	#Train	#Test
1	7871	2608
2	7834	2645
3	7643	2836
4	7754	2725
5	7889	2590

**Table 11:** Comparison of classification accuracy across different spectral features and methods without window overlap during feature extraction.

Feature	Method	Accuracy (mean ± std) @time-train (s)					Avg.	p-value	MFLOPs	#Params(M)
		1	2	3	4	5				
STFT	ResNet18	69.01±1.14 @853.7s	**70.23** ± **1.80** @856.3s	**69.68** ± **1.46** @855.2s	**78.01** ± **1.47** @859.9s	**72.98** ± **1.12** @868.3s	**71.92**	0.320	2178.02	11.17
	RCMoE-b.	68.32 ± 1.29 @1056.0s	68.37 ± 1.41 @1060.2s	68.25 ± 1.40 @1006.5s	77.47 ± 1.32 @1052.7s	72.18 ± 1.43 @1057.6s	70.86	0.595	2178.03	11.19
	CFTAnet	**69.34** ± **1.13** @454.3s	69.41 ± 0.93 @457.3s	68.46 ± 0.96 @423.2s	74.18 ± 1.02 @446.1s	69.04 ± 1.01 @456.8s	70.08	0.036	210.06	**0.55**
	FCResNet5	68.46 ± 0.95 **@187.0s**	69.98 ± 0.74 **@189.5s**	69.35 ± 0.66 **@184.6s**	77.29 ± 0.41 **@176.0s**	71.39 ± 0.50 **@188.8s**	71.29	–	**98.064**	0.65
Mel	ResNet18	68.17 ± 1.38 @862.2s	69.50 ± 1.45 @858.0s	**69.18** ± **1.23** @859.0s	**76.93** ± **0.82** @856.0s	**74.22** ± **0.94** @856.2s	**71.6**	0.223	2178.02	11.17
	RCMoE-b.	67.03 ± 1.73 @1056.2s	66.83 ± 3.49 @1150.8s	68.10 ± 1.95 @1007.8s	76.57 ± 1.08 @1054.6s	73.86 ± 0.88 @1056.2s	70.48	0.752	2178.03	11.19
	CFTAnet	68.06 ± 1.05 @442.4s	68.66 ± 1.30 @448.2s	66.23 ± 0.70 @412.0s	74.04 ± 1.15 @413.7s	68.35 ± 0.95 @443.1s	69.07	0.010*	210.06	**0.55**
	FCResNet5	**68.32** ± **1.20** ** @178.1s**	**70.10** ± **1.63** **@184.4s**	67.98 ± 1.06 **@168.9s**	76.84 ± 0.69 **@174.6s**	70.47 ± 0.82 **@187.4s**	70.74	-	**98.064**	0.65
CQT	ResNet18	66.68 ± 1.16 @358.6s	65.61 ± 2.10 @354.0s	64.20 ± 0.70 @354.3s	69.64 ± 1.78 @354.5s	**67.66** ± **0.98** @354.8s	66.76	0.310	769.26	11.17
	RCMoE-b.	68.33 ± 1.52 @544.4s	63.69 ± 2.98 @526.4s	64.18 ± 0.74 @504.6s	68.28 ± 1.26 @508.2s	67.21 ± 1.93 @538.1s	66.33	0.086	769.27	11.19
	CFTAnet	66.50 ± 1.27 @203.2s	65.28 ± 1.74 @204.4s	64.68 ± 1.25 @182.1s	67.56 ± 1.21 @204.5s	65.56 ± 1.72 @208.5s	63.92	0.003*	72.69	0.51
	FCResNet5	**67.03** ± **0.87** **@145.6s**	**65.93** ± **1.09** **@151.0s**	**64.59** ± **0.96** **@129.3s**	**71.66** ± **1.07** **@134.2s**	67.13 ± 0.96 **@137.5s**	**67.27**	–	**51.75**	**0.35**
Gamma -tone	ResNet18	62.47 ± 1.68 @858.0s	63.29 ± 1.42 @857.2s	59.32 ± 1.10 @865.0s	**69.75** ± **0.98** @857.8s	**67.06** ± **1.03** @910.9s	64.38	0.001*	2178.02	11.17
	RCMoE-b.	63.32 ± 1.36 @1079.9s	60.16 ± 2.92 @1057.3s	60.53 ± 0.62 @1014.4s	69.15 ± 1.23 @1049.9s	65.97 ± 1.22 @1066.0s	63.83	0.000*	2178.03	11.19
	CFTAnet	61.89 ± 0.81 @451.4s	63.54 ± 1.01 @437.1s	61.91 ± 1.14 @411.2s	64.34 ± 1.26 @409.3s	64.08 ± 1.16 @449.7s	63.15	0.000*	210.06	**0.55**
	FCResNet5	**65.73** ± **1.27** **@193.0s**	**66.49** ± **1.29** **@184.3s**	**64.59** ± **0.82** **@170.7s**	68.18 ± 1.02 **@176.3s**	66.93 ± 1.17 **@191.6s**	**66.38**	–	**98.064**	0.65

*Indicates *p* < 0.0167 (Bonferroni-corrected), denoting statistical significance.

**Table 12:** Comparison of classification accuracy across different spectral features and methods with 50% window overlap during feature extraction.

Feature	Method	Accuracy (mean ± std) @time-train (s)					Avg.	p-value	MFLOPs	#Params(M)
		1	2	3	4	5				
STFT	ResNet18	**72.55**±**1.36** @656.9s	69.41±2.29 @672.5s	**70.15**±**1.30** @656.5s	**79.06**±**0.46** @702.0s	**73.44**±**1.36** @867.7s	**72.92**	0.0511	4288.81	11.17
	RCMoE-b.	68.30±1.69 @792.1s	67.36±2.77 @776.7s	68.13±1.63 @751.8s	78.32±1.59 @770.2s	71.98±1.55 @825.1s	70.82	0.017	4288.82	11.19
	CFTAnet	68.71±0.66 @615.3s	**70.75**±**1.22** @585.8s	68.20±1.09 @580.1s	74.74±0.98 @573.1s	68.29±1.29 @629.0s	70.14	0.884	411.65	**0.55**
	FCResNet5	70.12±0.72 **@428.8s**	69.63±1.21 **@475.8s**	69.01±0.75 **@452.7s**	77.57±0.57 **@427.2s**	71.44±0.68 **@434.5s**	71.55	–	**196.12**	0.65
Mel	ResNet18	68.72±1.76 @619.2s	70.14±1.61 @686.7s	**69.13**±**1.43** @656.7s	**77.34**±**1.06** @648.1s	**73.75**±**1.03** @726.9s	**71.82**	0.000*	4288.81	11.17
	RCMoE-b.	67.02±1.45 @1015.1s	65.98±3.50 @795.6s	68.11±1.57 @779.0s	76.25±1.15 @782.6s	71.91±1.95 @761.3s	69.45	0.004*	4288.82	11.19
	CFTAnet	68.14±1.57 @593.0s	69.30±0.92 @582.9s	66.56±1.03 @550.9s	74.05±1.04 @547.1s	68.41±1.06 @568.6s	69.29	0.027	411.65	**0.55**
	FCResNet5	**69.03**±**1.17** **@403.9s**	**70.54**±**1.43** **@452.3s**	67.73±1.05 **@478.0s**	76.75±0.45 **@421.8s**	70.81±0.77 **@435.6s**	70.97	–	**196.12**	0.65
CQT	ResNet18	**67.60**±**1.28** @306.0s	65.52±1.60 @315.7s	64.02±1.17 @298.3s	69.28±1.32 @303.8s	**68.12**±**1.53** @314.6s	66.91	0.000*	1514.76	11.17
	RCMoE-b.	66.22±1.66 @369.1s	63.98±1.30 @358.9s	63.02±1.93 @329.5s	68.31±1.77 @313.9s	67.01±1.49 @322.0s	65.71	0.016*	1514.77	11.19
	CFTAnet	66.04±2.16 **@282.6s**	65.68±1.66 **@279.7s**	63.89±1.18 **@271.1s**	68.71±1.89 @302.2s	65.59±1.46 @308.9s	65.98	0.225	142.23	0.51
	FCResNet5	66.10±0.28 @305.1s	**66.00**±**0.54** @294.8s	**64.06**±**1.12** @283.6s	**71.62**±**0.98** **@254.0s**	67.14±1.09 **@296.5s**	**66.98**	-	**103.51**	**0.35**
Gamma -tone	ResNet18	65.82±1.37 @668.8s	64.88±2.37 @684.5s	60.93±1.24 @657.3s	**70.57**±**0.97** @679.1s	66.76±0.91 @683.0s	65.39	0.000*	4288.81	11.17
	RCMoE-b.	63.21±1.88 @794.8s	60.74±2.07 @753.7s	59.70±0.89 @704.1s	68.72±1.20 @752.6s	66.04±1.23 @812.4s	63.68	0.013*	4288.82	11.19
	CFTAnet	62.55±1.67 @599.7s	64.11±1.01 @529.4s	61.86±1.58 @518.6s	65.35±1.31 @522.8s	64.06±1.02 @559.4s	63.59	0.054	411.65	**0.55**
	FCResNet5	**66.10**±**0.66** **@408.7s**	**66.95**±**1.11** **@413.2s**	**66.16**±**1.17** **@415.0s**	68.79±0.66 **@429.1s**	**66.90**±**0.76** **@405.3s**	**66.98**	–	**196.12**	0.65

* indicates p < 0.0167 (Bonferroni-corrected), denoting statistical significance.

As shown in Tables 11 and 12, we present the classification performance of FCResNet5 compared to three representative baselines under 0% and 50% window overlap settings. Columns 3 to 7 report the accuracy (mean ± std over 10 trials) and training time for each of the five dataset folds using four types of spectral features. Column 8 summarizes the average accuracy across all folds, while Column 9 provides the p-values from two-sample t-tests between FCResNet5 and other competing methods under each feature. The last two columns display the model complexity in terms of MFLOPs and parameter count, offering a complete view of model efficiency and cost.

The results in Tables 11 and 12 reveal that ResNet18 performs best under STFT and Mel inputs, while FCResNet5 consistently ranks second. However, when using CQT and Gamma-tone features, FCResNet5 achieves the highest accuracy across both overlap settings, outperforming all baseline models. For instance, with no overlap, FCResNet5 achieves 66.38 on CQT and 66.98% on Gamma-tone, surpassing ResNet18 by 1.39% and 2.63%, respectively. Under 50% overlap, this advantage is maintained, with FCResNet5 reaching 66.98% and 66.90% on CQT and Gamma-tone, respectively.

While performance differences on STFT and Mel are relatively small, the consistent advantage of FCResNet5 on CQT and Gamma-tone demonstrates its adaptability across diverse input features. Given the limited performance gap and the consistent feature extraction settings, these variations are more likely attributed to statistical fluctuations rather than systematic feature dependence.

In addition to accuracy, FCResNet5 maintains significant efficiency benefits. It offers a substantial reduction in the number of parameters compared to ResNet18 and RCMoE-balance, with MFLOPs reduced by over 90% and shorter training times across all features. These results confirm that FCResNet5 achieves competitive performance while substantially reducing computational cost, making it a strong candidate for deployment in resource-constrained environments.

To assess performance differences, we conduct independent two-sample t-tests (Bonferroni-corrected, $p < 0.0167$) to compare FCResNet5 with baseline models under both non-overlap and 50% overlap conditions. Under the non-overlap setting, FCResNet5 shows significant advantages over ResNet18 and RCMoE-balance under the Gamma-tone feature ($p < 0.001$), while differences under STFT, Mel, and CQT are not statistically significant ($p > 0.0167$) despite variations in average accuracy. Under the 50% overlap setting, despite FCResNet5 showing a marginally lower average accuracy than ResNet18 under the Mel feature, the difference is statistically significant. For STFT, although ResNet18 again yields higher mean accuracy, the difference remains insignificant ($p > 0.0167$). These results indicate that while FCResNet5 offers competitive or superior performance across various scenarios, its improvements are feature-dependent and not always statistically significant. This underscores its practical value in resource-constrained environments, where balancing accuracy and efficiency is critical.

**Fig. 12:**
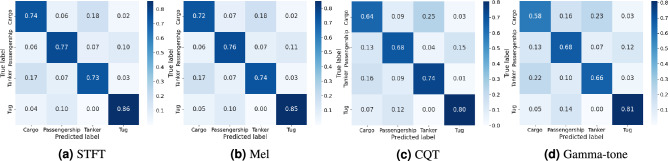
Confusion matrices of FCResNet5 using: (**a**) STFT, (**b**) Mel, (**c**) CQT, and (**d**) Gamma-tone on the test set.

To further interpret the classification behavior, Fig. 12 presents the normalized confusion matrices of FCResNet5 using the four different spectral features. These matrices reflect the average performance over 10 repeated runs on the test set. Among the four target classes, Tug is generally recognized with the highest recall across all feature types. STFT provides the most balanced classification, yielding strong performance for Passengership (0.77), while Mel leads in accuracy for the Cargo class (0.74). In contrast, CQT and Gamma-tone representations result in relatively higher inter-class confusion, especially for Cargo and Tanker. For instance, under Gamma-tone, the recall for Cargo and Tanker drops to 0.58 and 0.66, respectively, reflecting a noticeable decline in discriminability.

#### Robustness Evaluation under Different SNR Conditions

**Fig. 13:**
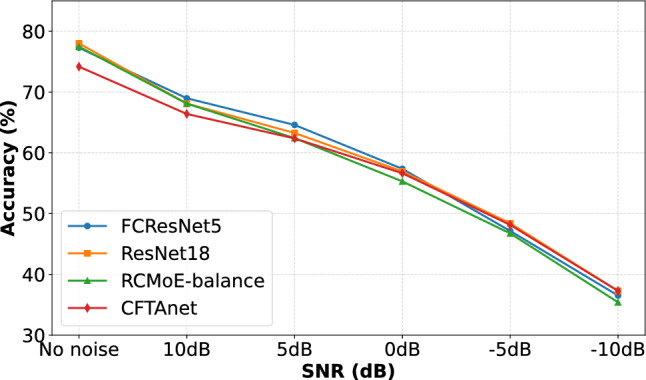
Classification accuracy of four models under different SNR levels.

To evaluate the robustness of different models in noisy environments, we simulate additive Gaussian noise at various SNR levels: clean (no noise), 10 dB, 5 dB, 0 dB, $-5$ dB, and $-10$ dB. Noise is added directly to the raw audio signals before feature extraction. We compare our proposed model FCResNet5 with a standard ResNet18 baseline, as well as two competitive methods from the literature: RCMoE-balance and CFTAnet. Each setting is evaluated over 10 independent runs, and we report the average classification accuracy across these runs. Figure 13 illustrates the classification performance of different models under varying SNR conditions. As the noise level increases, all models exhibit a consistent decline in accuracy, indicating sensitivity to noise interference. FCResNet5 achieves the highest accuracy when the SNR is above 0 dB, suggesting its suitability for clean to moderately noisy environments. However, as the SNR drops below 0 dB, its performance deteriorates more noticeably, and CFTAnet slightly surpasses it in extreme noise conditions (e.g., −5 dB and −10 dB). These results indicate that while all models are vulnerable to severe noise, the overall robustness across models remains comparable. Enhancing low-SNR resilience without sacrificing model efficiency remains an important direction for future investigation.

## Conclusion

In this paper, we propose FCResNet5, a lightweight and efficient deep learning model tailored for SRN classification. The design of FCResNet5 is guided by three core principles: focusing on the dominant frequency band of SRN signals, leveraging frequency channelization to enhance spectral resolution, and adopting a compressed descending channel structure to reduce computational cost.

We first examine input representations and find that time-frequency features consistently offer better discriminative power than non-time-frequency ones. Ablation studies confirm the effectiveness of frequency band selection, channelization, and the descending structure. Further comparisons across spectral features and SNR conditions demonstrate that FCResNet5 achieves a favorable trade-off between accuracy and efficiency.

In future work, we plan to explore optimized architectures to improve classification while preserving simplicity, enhance performance under extremely low SNR conditions, and adopt automated compression strategies to better balance accuracy and efficiency.

## Data Availability

The datasets analyzed during the current study are available for download at https://github.com/irfankamboh/DeepShip repository. (Keeping in view the limitation of file size and space on github directory, only a part of dataset is uploaded on this repository. The complete dataset can be downloaded by following the information provided on the same repository web page).
